# Physiological and structural adjustments of two ecotypes of *Platanus orientalis* L. from different habitats in response to drought and re-watering

**DOI:** 10.1093/conphys/coy073

**Published:** 2018-12-20

**Authors:** Violeta Velikova, Tsonko Tsonev, Massimiliano Tattini, Carmen Arena, Sashka Krumova, Dimitrina Koleva, Violeta Peeva, Svetoslav Stojchev, Svetla Todinova, Luigi Gennaro Izzo, Cecilia Brunetti, Miroslava Stefanova, Stefka Taneva, Francesco Loreto

**Affiliations:** 1 Institute of Plant Physiology and Genetics, Bulgarian Academy of Sciences, Acad. G. Bonchev Str. bl. 21, Sofia, Bulgaria; 2 Institute of Biophysics and Biomedical Engineering, Bulgarian Academy of Sciences, Acad. G. Bonchev Str., bl. 21, Sofia, Bulgaria; 3 Institute for Sustainable Plant Protection, Department of Biology, Agriculture and Food Sciences, The National Research Council of Italy (CNR), Sesto Fiorentino (Florence), Italy; 4 Department of Biology, University of Naples Federico II, Via Cinthia, Naples, Italy; 5 Faculty of Biology, Sofia University, Sofia, Bulgaria; 6 Department of Agricultural Sciences, University of Naples Federico II, Via Università 100, Portici, Italy; 7 Department of Biology, Agriculture and Food Sciences, Trees and Timber Institute, The National Research Council of Italy (CNR), Sesto Fiorentino (Florence), Italy; 8 Department of Biology, Agriculture and Food Sciences, The National Research Council of Italy (CNR), Rome, Italy

## Abstract

*Platanus orientalis* covers a very fragmented area in Europe and, at the edge of its natural distribution, is considered a relic endangered species near extinction. In our study, it was hypothesized that individuals from the edge of the habitat, with stronger climate constrains (drier and warmer environment, Italy, IT ecotype), developed different mechanisms of adaptation than those growing under optimal conditions at the center of the habitat (more humid and colder environment, Bulgaria, BG ecotype). Indeed, the two *P. orientalis* ecotypes displayed physiological, structural and functional differences already under control (unstressed) conditions. Adaptation to a dry environment stimulated constitutive isoprene emission, determined active stomatal behavior, and modified chloroplast ultrastructure, ultimately allowing more effective use of absorbed light energy for photochemistry. When exposed to short-term acute drought stress, IT plants showed active stomatal control that enhanced instantaneous water use efficiency, and stimulation of isoprene emission that sustained photochemistry and reduced oxidative damages to membranes, as compared to BG plants. None of the *P. orientalis* ecotypes recovered completely from drought stress after re-watering, confirming the sensitivity of this mesophyte to drought. Nevertheless, the IT ecotype showed less damage and better stability at the level of chloroplast membrane parameters when compared to the BG ecotype, which we interpret as possible adaptation to hostile environments and improved capacity to cope with future, likely more recurrent, drought stress.

## Teaser

*Platanus orientalis* adapts to habitat-specific micro-environmental conditions through functional and structural modifications. Drier environments stimulate constitutive isoprene emission, determine active stomatal behavior and the formation of thicker grana stacks, which could enhance the performance of *P. orientalis* under future stress conditions.

## Introduction

Models forecast more recurrent and intense heat waves, drought and flooding events as a consequence of climate change caused by rising CO_2_ concentration and temperature on the Earth’s surface (IPCC, 2014). Plants cope with changing climate through evolutionary adaptation and phenotypic plasticity (Valladares et al., 2007; Hoffmann and Sgro, 2011; Nicotra et al., 2010; Becklin et al., 2016), involving physiological, metabolic and/or structural adjustments, often resulting in new ecotypes (Kooyers, 2015). However, extreme and fast episodes of climate change could challenge the adaptive capacity of plants with reduced plasticity (Stockwell et al., 2003; Leimu et al., 2010).

Climate change-exacerbated drought is expected to have a major negative impact on crops and native plants (Blum, 1996; Theurillat and Guisan, 2001). Drought primarily affects photosynthesis, the key process of primary metabolisms and productivity (for reviews see Chaves et al., 2003, 2009; Flexas et al., 2004; Lawlor and Tezara, 2009). Drought-induced reduction of photosynthesis is attributed to stomata closure and the consequent diffusive limitation of CO_2_ entry, impaired photochemistry and enhanced-metabolite fluxes into defense compounds (Chaves et al., 2003). Stomata also regulate water loss, and stomatal control is critical to plant adaptation to arid environment, and an important component of plant water saving strategies, including hydro-passive (driven by leaf water potential) and hydro-active (e.g. ABA-driven) stomatal control (Raschke, 1975a; Maroco et al., 1997; Chaves et al., 2003; Chaves and Oliveira, 2004; David et al., 2007).

Drought could be particularly deleterious for the performance of fast-growing plants, which are hygrophilous in their nature, and have a poor stomatal control over water loss (Ngugi et al., 2004; Silim et al., 2009). In our study, two populations of oriental plane (*Platanus orientalis*) located in Italy and Bulgaria were compared. *P. orientalis* is a fast-growing deciduous tree, whose habitat spans south-east European and south-west Asian warm riparian forests. Southern Italy (Campania, Apulia and Sicily) (Rosati et al., 2015) is the westernmost limit of *P. orientalis* distribution (Caruso et al., 2008) where the species is now endangered and near extinction (Barstow and Rivers, 2017). In Bulgaria, *P. orientalis* reaches the northernmost limit in its natural distribution (Grueva and Zhelev, 2011) where plants adapted to cooler and more humid environments. Thus, it is likely that individuals growing at the edge of the habitat, where a changing climate is a powerful constraint, developed adaptation mechanisms different than plants which thrive in more suitable environments (Finni et al., 2017). Recently, we have demonstrated that two *Arundo donax* ecotypes originating from stands with different climate showed different physiological and metabolic features when grown under well-watered conditions, and these phenotypic differences determined different drought-stress responses (Ahrar et al., 2017).

In fast growing and hygrophilous tree species, isoprene emission is a common trait (Loreto and Fineschi, 2015), and *Platanus* species are strong isoprene emitters (Kesselmeier and Staudt, 1999; Velikova et al., 2006; Loreto and Fineschi, 2015). Isoprene is considered to be a good antioxidant per se (Vickers et al., 2009; Velikova et al., 2011), and a proxy of other antioxidants that are synthesized through the same biochemical pathway, e.g. xanthophylls (Tattini et al., 2015). It was demonstrated that the suppression of isoprene emission through genetic manipulation or chemical inhibition negatively affects thylakoid membrane function and integrity (Velikova et al., 2011, 2015). However, isoprene biosynthesis is metabolically and energetically expensive for plants (Sharkey and Yeh, 2001), and the trait might have been lost under optimal conditions, or replaced by more suitable mechanisms in very stressful environments. It was suggested that isoprene facilitates C3 photosynthesis under optimal conditions (Pollastri et al., 2014), or helps plants overcome transient and mild stresses (Loreto and Fineschi, 2015). It was also postulated that the isoprene emission trait must have evolved independently in the major lineages of land plants by parallel evolution (Sharkey et al., 2013); or that *IspS* genes can undergo repeated gain and loss at the family and even genus level thanks to the hypothetically low number of amino acidic mutations which may be sufficient for evolution (Monson et al., 2013). Isoprene emission might have evolved in hygrophilous species as a first mechanism of adaptation to terrestrial life (Loreto et al., 2014) and could provide new information about the distribution of genetic diversity, and local capacity of plant adaptation (Aitken et al., 2008). We hypothesized that ecotypes growing under different environmental conditions developed a different capacity to emit isoprene, which in turn produces ecotype differences in chloroplast fine structure, physical properties of the thylakoid membranes and overall functionality of the photosynthetic apparatus (Velikova et al., 2015). Specifically, the capacity to emit isoprene might allow *Platanus* ecotypes to survive at the edge of the habitat of the species, and may therefore constitute a trait serving for applied plant conservation purposes. We tested this hypothesis exposing the two contrasting ecotypes of oriental plane to drought stress and recovery from stress.

## Material and methods

### Plant material and growth conditions

*P. orientalis* seeds were collected from native populations in Francavilla di Sicilia, Sicily, Italy (IT) (37.541976′N, 15.082318′E; mean summer temperature ~24°C and mean summer rainfalls ~14 mm) and in Kresna, Bulgaria (BG) (41.440800′N, 23.082929′E; mean summer temperature ~23°C and mean summer rainfalls ~43 mm) (https://en.climate-data.org/location/194719/). According to the Köppen–Geiger climate classification, the climate in Francavilla di Sicilia is classified as Csa (hot dry-summer), and in Kresna as Cfa (humid mild temperate) (https://en.climate-data.org/location/194719/, http://hanschen.org/koppen/).

Seeds of the two ecotypes were germinated and plant seedlings were grown in a climate chamber with the following controlled conditions: light intensity (PPFD) 350 μmol m^−2^ s^−^^1^, day/night temperature 25/20°C ± 2°C, relative humidity 65–70%, photoperiod 14 h and ambient CO_2_ concentration 400 μmol mol^−^^1^. Plants were grown under these conditions for 4 months. During this period, plants were regularly watered to keep the pots to full water capacity, and were fertilized every two weeks with full-strength Hoagland solution to supply mineral nutrients at free access rates.

The first set of measurements was performed in well-watered saplings (controls). Then, plants were divided in two groups of 28 plants each. Fourteen plants of each ecotype were kept under well-watered conditions throughout the experimental period, to assess possible age effect, while the other 14 plants were subjected to drought. No changes due to aging were detected in well-watered IT and BG plants, and these measurements are therefore not shown. The plants undergoing drought stress were further divided in two groups: eight plants were used for non-destructive measurements and six for destructive measurements, as specified below.

Drought stress was initiated by stopping watering, and the pot water content was daily calculated using the fraction of transpirable soil water (FTSW, %) parameter (Brilli et al., 2007). The second set of measurements was performed after 6–7 days of drought stress, when the FTSW reached 28 ± 2%. Then, plants were re-watered to full pot capacity, and a third set of measurements were performed after 7 days, when FTSW was 90%. Third and fourth fully expanded leaves from the apex were used for all analyses.

### Plant water status

The leaf relative water content (RWC) was calculated as [(FW−DW)/(TW−DW)] x 100, where FW is the fresh weight, DW is the dry weight after drying the leaf at 80°C for 24 h, and TW is the turgid weight of the leaf reached after keeping the leaf in distilled water for 24 h. Leaf RWC was determined in well-watered (control), drought-stressed and re-watered plants.

### Gas exchange and chlorophyll fluorescence measurements

Leaf photosynthetic gas exchange was measured by a portable gas-exchange system (LCpro+, ADC BioScientific, UK). The middle part of the leaf was clamped into the 6.25-cm^2^ gas-exchange system cuvette and exposed to a constant flow (300 μmol s^−^^1^) of synthetic air (79% N_2_, 21% O_2_ and 400 μmol mol^−^^1^ CO_2_). All measurements were carried out at 25 ± 1°C leaf temperature and 800 μmol m^−^^2^ s^−^^1^ photosynthetic photon flux density (PPFD) at the leaf level. The relative humidity in the leaf chamber was set at 45–50%. The intrinsic water use efficiency (iWUE) was calculated as a ratio between photosynthesis (*A*_n_) and stomatal conductance (*g*_s_). The CO_2_ diffusing into the intercellular spaces (*C*_i_) was calculated using the formulation of von Caemmerer and Farquhar (1981) with the gas-exchange system software. To assess the physiological behavior of stomata, the response of *g*_s_ to increasing [CO_2_] in the range of 50–1800 μmol mol^−1^ was analyzed. At each [CO_2_] step all gas-exchange parameters were recorded after reaching a steady-state, usually 5–10 min after the change in [CO_2_].

Chlorophyll a fluorescence was measured by the IMAGING-PAM M-series chlorophyll fluorometer (Heinz Walz GmbH, Effeltrich, Germany). Plants were dark-adapted for 30 min prior to the determination of minimum (*F*_o_) and maximum (*F*_m_) fluorescence and then a saturating light pulse of 0.8 s with a PPFD > 3000 μmol photons m^−^^2^ s^−^^1^ was applied. The maximum quantum yield of photosystem II (PSII) was calculated as *F*_v_/*F*_m_ = (*F*_m_*−F*_o_)/*F*_m_. Leaves were then exposed to actinic light (400 μmol m^−^^2^ s^−^^1^ PPFD) in order to obtain chlorophyll fluorescence in light-adapted state. After reaching the steady-state fluorescence (*F*_s_), the application of a second saturating pulse under actinic light conditions allowed to determine the PSII quantum efficiency in illuminated leaves [*Φ*_PSII_ = (*F*′_m_−*F*_s_)/*F*′_m_], where *F*_m_′ is the maximum fluorescence in light-adapted state (Genty et al., 1989). The non-photochemical quenching (NPQ) was defined according to the equation NPQ = (*F*_m_−*F*_m_′)/*F*_m_′ (Bilger and Björkman, 1991).

### Volatile organic compounds analysis

The emissions of isoprene and hexenal, the latter being an indicator of membrane damage (Loreto et al., 2006), were detected by gas chromatography-mass spectrometry (GC–MS) as reported in Beckett et al. (2012). Shortly, volatile organic compounds (VOCs) were collected under the same conditions used for measuring the photosynthetic gas-exchange parameters by directing part of the air flowing out of the leaf cuvette into a silicosteel cartridge packed with 200 mg of Tenax (Markes International Ltd, Llantrisant, UK). The cartridges were analyzed with a Perkin Elmer Clarus 580 GC coupled with a Clarus 560 MS detector and a thermal desorber TurboMatrix (Perkin Elmer Inc., Waltham, MA, USA) as detailed in Velikova *et al.* (2016). The GC–MS system was calibrated using gas standard for target compounds. The compounds were identified via the National Institute of Standards and Technology (NIST) library provided with the GC–MS ChemStation software (Agilent Technologies and Perkin Elmer). GC peak retention time was substantiated by analysis of parent ions and main fragments on the spectra. The concentration of each volatile compound was calculated by comparison with the peak area of the gaseous standard.

### Isolation of thylakoid membranes

Thylakoid membranes from *P. orientalis* L. were isolated according to the protocol of Harrison and Melis (1992) with minor modifications. Leaf samples were collected at the end of dark period, when starch level was minimal. Fully expanded leaves (third and fourth node from the apex) were homogenized in a medium containing 50 mM Tricine (pH 7.8), 5 mM MgCl_2_, 10 mM NaCl, 400 mM sucrose (isolation buffer), filtered through cheese cloth and centrifuged at 4600 x g. The chloroplasts containing pellet was resuspended in a hypotonic medium containing 50 mM Tricine (pH 7.8), 5 mM MgCl_2_, 10 mM NaCl and centrifuged at 5000 × g. The thylakoid fraction was finally resuspended in the isolation buffer, supplemented with 30% glycerol (v/v) and stored at −20°C. Before each measurement, the membranes were washed twice in the measuring buffer containing 20 mM Tricine (pH 7.8), 250 mM sorbitol and 5 mM MgCl_2_, the chlorophyll (Chl) concentration was determined according to Arnon (1949) and standardized to 0.7 mg Chl ml^−^^1^ and 20 μg Chl ml^−^^1^ for calorimetric and spectroscopic measurements, respectively. Freshly-isolated thylakoid membranes were used for further differential scanning calorimetry (DSC) and merocyanine 540 fluorescence measurements.

### Differential scanning calorimetry measurements

DSC profiles (thermograms) were measured using a DASM4 high-sensitivity scanning microcalorimeter (Biopribor, Pushchino, Russia). A buffer–buffer scan was subtracted from each experimental DSC scan, followed by subtraction of a linear-baseline fit. The samples were scanned in the range 30°C–100°C at a heating rate of 1°C min^−^^1^. The data were analyzed with the Origin 6.0 software package (OriginLab Corporation, Northampton, MA, USA). The transition temperatures (*T*_m_) are defined as temperatures at the maximum of the excess heat capacity curves. The calorimetric enthalpy (Δ*H*_cal_) of the thermograms was estimated by integrating the total area under the excess heat capacity curve and the cooperativity (*T*_1/2_) was evaluated as the width at half height of the main transition.

### Merocyanine 540 fluorescence

The spectral characteristics of the lipophylic fluorescence marker merocyanin 540 (MC540) were used to analyze the physical state of the lipid matrix of thylakoid membranes. Excitation spectra of MC540 incorporated in thylakoid membranes were recorded with a Jobin Yvon JY3 spectrofluorometer by collecting the emission 590 nm upon light excitation in the range 450–575 nm, in 1 nm step and applying 10 nm emission and excitation slits. Prior to measurements, an aliquot of MC540 stock solution (1mM MC540 dissolved in ethanol) was added to thylakoid membrane suspension at final concentration of 0.2 μM and the samples were incubated for 20 min. In order to correct for the contribution of Chl fluorescence, excitation spectra of thylakoid membranes were recorded in the absence of MC540 and subtracted from the corresponding spectra obtained after the addition of the probe. All measurements were performed at 25°C.

### Thermoluminescence measurements

Thermoluminescence (TL) emission by leaf discs (diameter 10 mm) was measured with a home-made apparatus described in detail in Zeinalov and Maslenkova (1996). In brief, freshly excised discs from middle part of the leaf with the exception of the veins were placed on the sample holder aluminum surface at 20°C and covered with a plexiglas window. After cooling the sample by liquid nitrogen to 1°C the samples were illuminated with 1, 2 or 3 saturating (4J) single turnover xenon flashes (10 μs half-band, 1 Hz frequency) of white light. Then the sample was warmed up to 70°C at a 0.5-°C s^−^^1^ heating rate. Temperature of the sample was measured with a tiny thermocouple, inserted in the sample holder. Luminescence was detected by HR943-02 photomultiplier (Hamamatsu Photonics, Japan).

The TL signals were read and registered by a computer using home-made software. The signals from data files were smoothed and the temperature maximum (*T*_max_) of the individual bands was determined after signal decomposition by using Origin 8.5 Multiple peak fit (OriginLab Corporation, Northampton, MA, USA).

### Leaf protein extraction and western blot analysis

Leaf protein extraction was carried out following the procedure of Wang et al. (2006). Leaf tissues (1 g), were fine grinded in a mortar under liquid nitrogen. The powder was suspended in 10% TCA/acetone solution, centrifuged at 16 000 × *g* for 3 min at 4°C and then washed first in methanol (80%) and after in acetone (80%). After drying (50°C for 10 min), the pellet was resuspended in 1:1 phenol (pH 8.0)/SDS buffer and centrifuged at 16 000 × *g* for 3 min. The upper phenol phase was treated with methanol containing 0.1 M ammonium acetate, stored overnight at −20°C and centrifuged again. The pellet was washed once with 100% methanol and once with 80% acetone and after resuspended in a SDS sample buffer.

The detection of D1 (PsbA) and Actin proteins was carried out by western blotting analysis. Proteins (5 μg) extracted from leaves together with PsbA and Actin protein standard (2 μg) were analyzed on 12% dodecyl sulfate-polyacrylamide gel electrophoresis (SDS-PAGE) and electrotransferred onto 0.2 μm pore-size nitrocellulose membrane (Bio-Rad Laboratories S.r.l., Segrate, Milano, Italy) at 100 V for 4 h at 4°C in the transfer buffer (tris-glicine, methanol, H_2_O). The nitrocellulose membrane was treated for 1 h with blocking solution (50 mM Tris–HCl, pH 8.0, 150 mM NaCl, 0.5% (v/v) Tween 20, TBS-T) and 5% (w/v) non-dry milk and incubated with anti-PsbA, hen polyclonal serum (Agrisera, 1:50000, v/v) and anti-ACT, rabbit polyclonal serum (Agrisera, 1:2500, v/v) for 1 h at room temperature in the same solution supplemented with 5% (w/v) non-dry milk.

The blot was then washed several times with buffer TBS-T and the binding antibodies detected using as secondary antibody goat anti-hen IgY horse radish peroxidase (HRP) conjugated (Agrisera, 1:50000, v/v) for D1 and anti-rabbit HRP conjugated antibody (Agrisera, 1:10000 v/v) for Actin. The immunorevelation of HRP reaction was performed using the kit for chemiluminescence (Westar Supernova, Cyanagen Srl, Bologna, Italy) by ChemiDoc System (Bio-Rad) (Arena et al., 2017).

Densitometry analysis was performed using ImageJ software (Rasband, WS, US NIH, Bethesda, Maryland, USA, 1997–2012). Each D1 protein band was normalized to the appropriate Actin band. Results were expressed in arbitrary units and referred to leaf dry weight.

### Transmission electron microscopy

Chloroplast ultrastructure was studied by transmission electron microscopy (TEM). Small leaf segments (1–2 mm^2^) were fixed in 3% (m/v) glutaraldehyde in 0.1 M sodium phosphate buffer (pH 7.4) and post-fixed in 1% (m/v) KMnO_4_ in the same buffer for 2 h at room temperature. After dehydration by increasing concentrations of ethyl alcohol (from 25% to 100%), the samples were embedded in Durcupan (Fluka, Buchs, Switzerland) and cross-sectioned with a Reichert-Jung (Wien, Austria) ultramicrotome. Observations were performed with a transmission electron microscope (JEOL1200 EX, Tokyo, Japan). At least 20 micrographs for each treatment were analyzed.

### Data analysis

Data shown represent the means ± SE of measurements on 28 different plants in total. The sample size of each measurement is reported in the corresponding figure legend. Data were subjected to one-way analysis of variance (ANOVA). Significant differences among means were estimated at the 5% (*P* < 0.05) level, using the Tukey’s test.

## Results

### Leaf RWC, chlorophyll fluorescence and photosynthetic gas exchange

**Figure 1:**
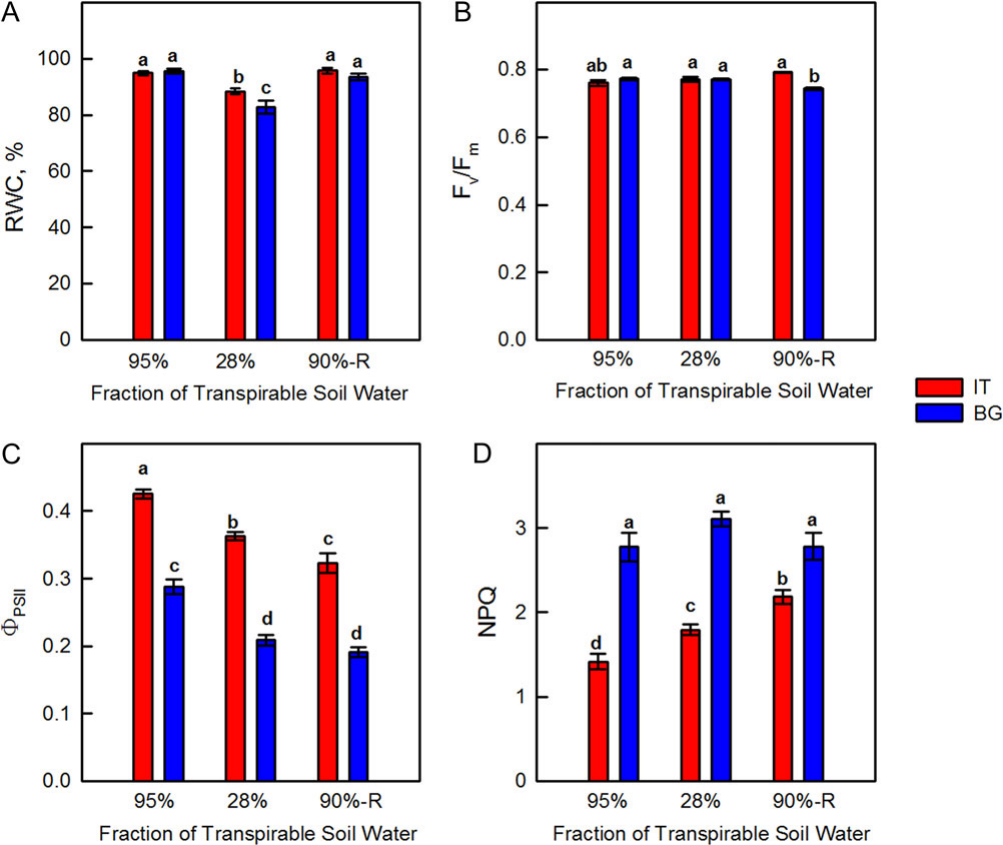
Leaf relative water content (RWC, Panel **A**), maximum quantum yield of PSII in dark-adapted plants (*F*_v_/*F*_m_, Panel **B**), PSII photochemical efficiency in light-adapted state (Φ_PSII_, Panel **C**) and non-photochemical quenching (NPQ, Panel **D**) in Italian (IT red bars) and Bulgarian (BG, blue bars) populations of *P. orientalis* plants measured under control conditions (95% FTSW), during drought (28% FTSW) and after re-watering (90%-R FTSW) treatments. Error bars indicate ± SE of the mean (*n* = 8). Data were subjected to one-way ANOVA followed by Tukey’s test and bars accompanied by different letters are statistically different (*P* < 0.05).

Under severe drought stress (28% FTSW) the leaf RWC was reduced to 82.7 ± 2.3 in BG and 88.4 ± 1.1 in IT *P. orientalis* leaves with respect to well-watered leaves (where RWC was close to 100%). Although moderate, this difference between ecotypes was statistically significant at *P* < 0.05 (Fig. 1A). Re-watering restored the original RWC of leaves of the two ecotypes.

The maximal efficiency of chlorophyll fluorescence in dark-adapted leaves (*F*_v_/*F*_m_) was not different between the two ecotypes before stress, and did not statistically change under drought. After re-watering, *F*_v_/*F*_m_ decreased by 14% in BG, whereas it increased by 17% in IT, and the difference between ecotypes was statistically significant (Fig. 1B).

The IT ecotype was characterized by a significantly higher efficiency of chlorophyll fluorescence in illuminated samples (*Φ*_PSII_) in comparison to BG, during the whole experiment (Fig. 1C). Drought stress caused reduction of *Φ*_PSII_ in both ecotypes, but the reduction was stronger in BG (-28%) than in IT (-15%). After re-watering, *Φ*_PSII_ further decreased in both BG and IT, as compared to the respective controls.

The NPQ of chlorophyll fluorescence was significantly lower in the IT than in the BG ecotype under control conditions (Fig. 1D). Drought stress stimulated NPQ in both ecotypes, and significantly more in IT. After re-watering, NPQ remained higher in IT, while it reached pre-stress level in the BG ecotype. In all cases, NPQ of the IT ecotype never reached the level of NPQ of the BG ecotype.

**Figure 2:**
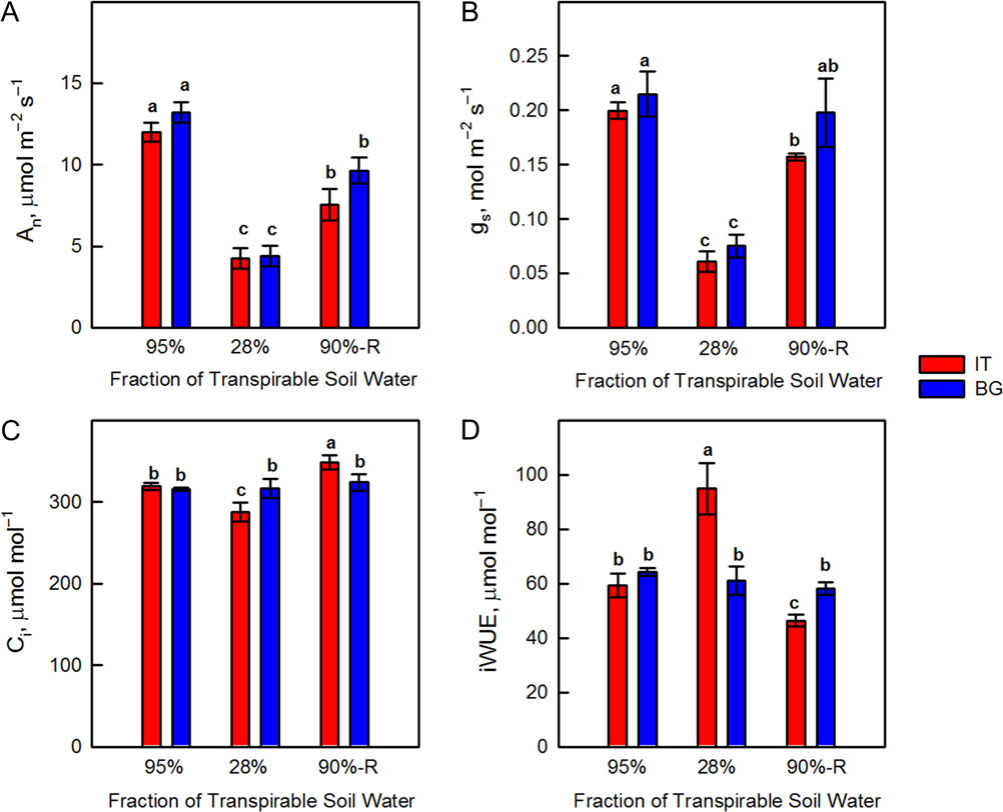
Net photosynthetic rate (*A*_n_, Panel **A**), stomatal conductance (*g*_s_, Panel **B**), intercellular [CO_2_] (*C*_i_, Panel **C**) and intrinsic water use efficiency (iWUE, Panel **D**) in Italian (IT, red bars) and Bulgarian (BG, blue bars) populations of *P. orientalis* plants measured under control conditions (95% FTSW), during drought (28% FTSW) and after re-watering (90%-R FTSW) treatments. Error bars indicate ± SE of the mean (*n* = 8). Data were subjected to one-way ANOVA followed by Tukey’s test and bars accompanied by different letters are statistically different (*P* < 0.05).

Photosynthesis (*A*_n_) was not different in the two ecotypes under control conditions. *A*_n_ was negatively affected by drought in both BG (-67%) and IT (-64%) ecotypes (Fig. 2A). This corresponded to a reduction in stomatal conductance to CO_2_ (*g*_s_) by 65% (BG) and 70% (IT) (Fig. 2B). Reduction of *g*_s_ was accompanied by a significant decrease in intercellular CO_2_ concentration (*C*_i_) in IT plants, while *C*_i_ was unaffected in the BG ecotype during the entire experimental period (Fig. 2C). As *A*_n_ and *g*_s_ decreased by a similar magnitude under drought stress, the iWUE did not significantly change in the BG ecotype. However, in the IT ecotype where *g*_s_ reduction outweighed the reduction of *A*_n_, iWUE increased by 40% under drought stress with respect to iWUE of controls and drought-stressed BG plants, indicating better control of stomatal closure over CO_2_ and water gas exchange (Fig. 2D). When FTSW reached 90%, after re-watering, an incomplete recovery of *A*_n_ was found in both IT and BG leaves (Fig. 2A). Photosynthesis of BG leaves reached 73% of the control value, while in IT *A*_n_ recovered to 63% of the corresponding controls. Slightly higher *A*_n_ in BG was correlated with better recovery of *g*_s_ in these plants compared to the IT ecotype (Fig. 2B). iWUE decreased in IT after re-watering and it was significantly lower than in re-watered BG leaves (Fig. 2D).

**Figure 3:**
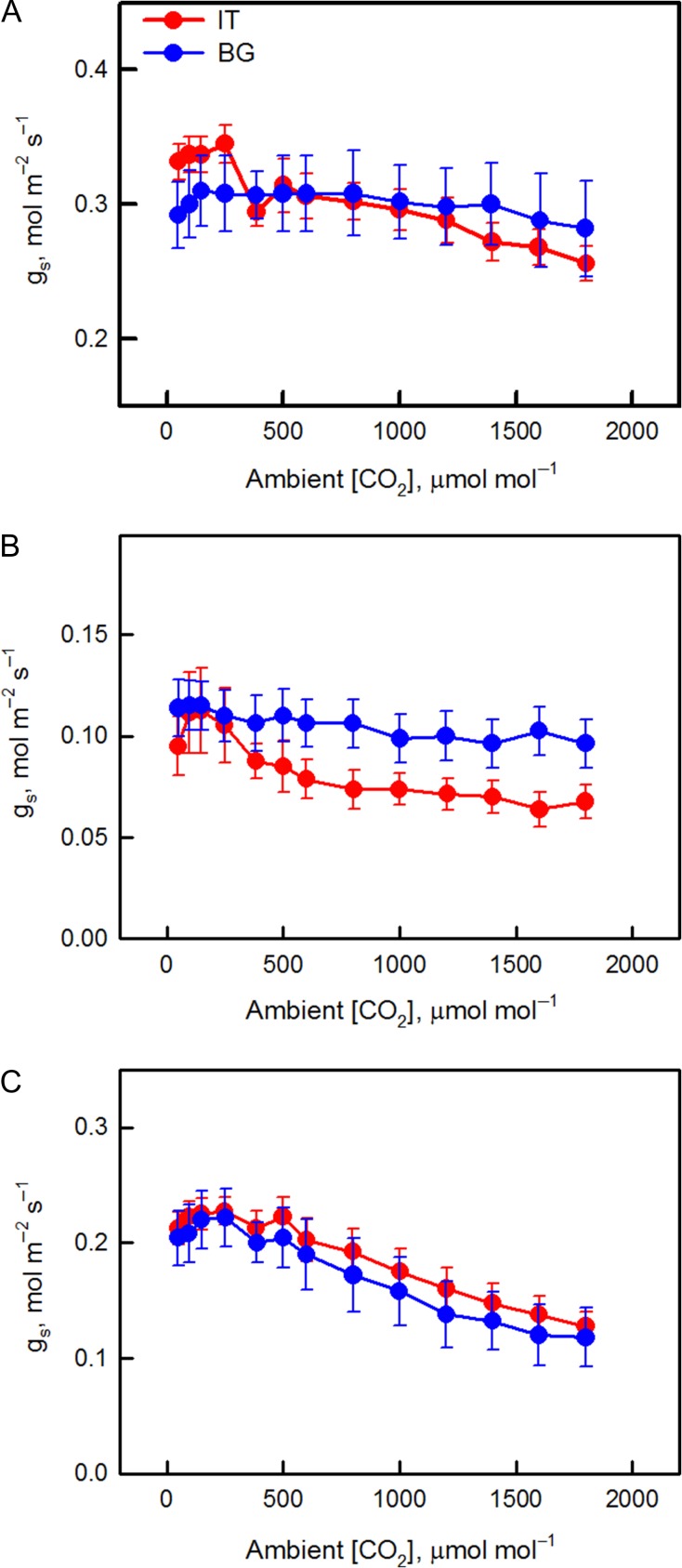
Response of stomatal conductance (*g*_s_) to increasing [CO_2_] of Italian (IT, red circle) and Bulgarian (BG, blue circle) *P. orientalis* plants under (**A**) control conditions (95% FTSW), (**B**) exposure to drought (28% FTSW) and (**C**) re-watering (90%-R FTSW). Error bars indicate SE of the mean (*n* = 8).

In order to further assess stomatal behavior, we analyzed the response of stomatal conductance to increasing [CO_2_] (Fig. 3). The BG ecotype showed a general lack of *g*_s_ response to [CO_2_] under control and drought conditions. The reduction of *g*_s_ from ambient to highest [CO_2_] was 10 (control) and 16% (drought-stress), indicating passive stomatal behavior (Fig. 3A). However, in the IT ecotype *g*_s_ decreased by 26% (control) and 40% (drought-stress) when comparing ambient and highest [CO_2_] (Fig. 3B), suggesting active regulation of stomatal behavior (Haworth et al., 2015). A similar, active stomatal response to [CO_2_] was observed in both IT and BG ecotypes when FTSW reached 90%, after re-watering (Fig. 3C), suggesting that drought stress sensitized stomata to [CO_2_] in BG plants (Raschke, 1975b).

### VOC emissions

**Figure 4:**
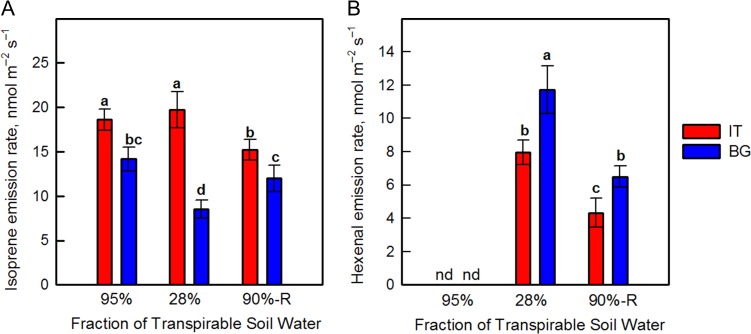
Isoprene (**A**) and hexenal (**B**) emission rate in Italian (IT red bars) and Bulgarian (BG, blue bars) populations of *P. orientalis* plants measured under control conditions (95% FTSW), during drought (28% FTSW) and after re-watering (90%-R FTSW) treatments. Error bars indicate ± SE of the mean (*n* = 8). Data were subjected to one-way ANOVA followed by Tukey’s test and bars accompanied by different letters are statistically different (*P* < 0.05).

Isoprene emission was significantly higher in the IT than in the BG ecotype under control conditions (Fig 4A). When plants were exposed to drought-stress isoprene emission was slightly stimulated in the IT ecotype, but decreased by 40% in the BG ecotype. After re-watering, isoprene emission of BG leaves increased to values similar to those measured in control plants, while in the IT ecotype isoprene emission decreased, reaching values lower than in controls. The emission of hexenal, which is a sensitive marker of membrane denaturation (Loreto et al., 2006), was undetectable in controls and considerably increased upon drought stress in the IT and especially in the BG ecotype (Fig. 4B). After re-watering, the hexenal emission decreased with respect to that observed during drought stress, but remained higher in the BG than in the IT ecotype.

### Chloroplasts ultrastructure

**Figure 5:**
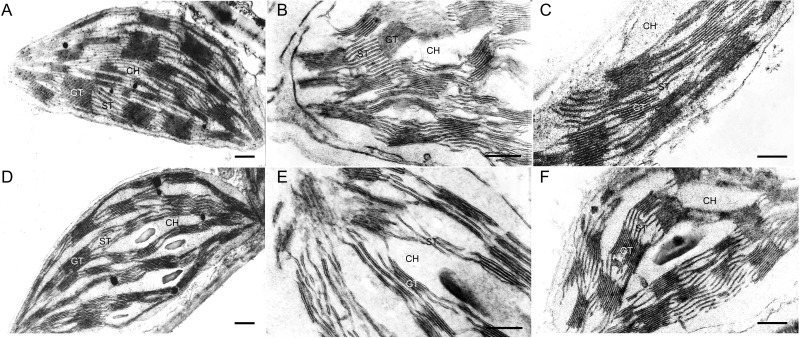
Representative electron micrographs of mesophyll chloroplasts in IT (**A**, **B**, **C**) and BG (**D**, **E**, **F**) ecotypes of *P. orientalis*. Chloroplast micrographs illustrate thylakoid membrane organization in control (95% FTSW) (A, D), drought-stressed (28% FTSW) (B, E) and re-watered (90% FTSW) (C, F) plants. Bars = 500 nm. CH, chloroplast; GT, grana thylakoids; ST, stroma thylakoids.

Thin segments obtained from the middle part of BG and IT leaves were subjected to TEM analysis (Fig. 5). The mesophyll chloroplasts of controls of both ecotypes had similar morphological characteristics, with a well-developed inner membrane system (Fig. 5A, D). However, IT chloroplasts showed higher stacked grana (~20–25 thylakoids) and more stroma thylakoids than the BG ecotype. The chloroplast membrane system was affected by drought in a similar way in both ecotypes as the stroma and part of the grana thylakoids were largely fragmented. However, these changes were more visible in the BG than in the IT chloroplasts (Fig. 5B, E). After re-watering, the fine structure of IT chloroplasts was similar to that of controls (Fig. 5C), while the membrane system of BG chloroplast only partially recovered its integrity (Fig. 5F).

### D1 content and physical properties of the thylakoid membranes

**Figure 6:**
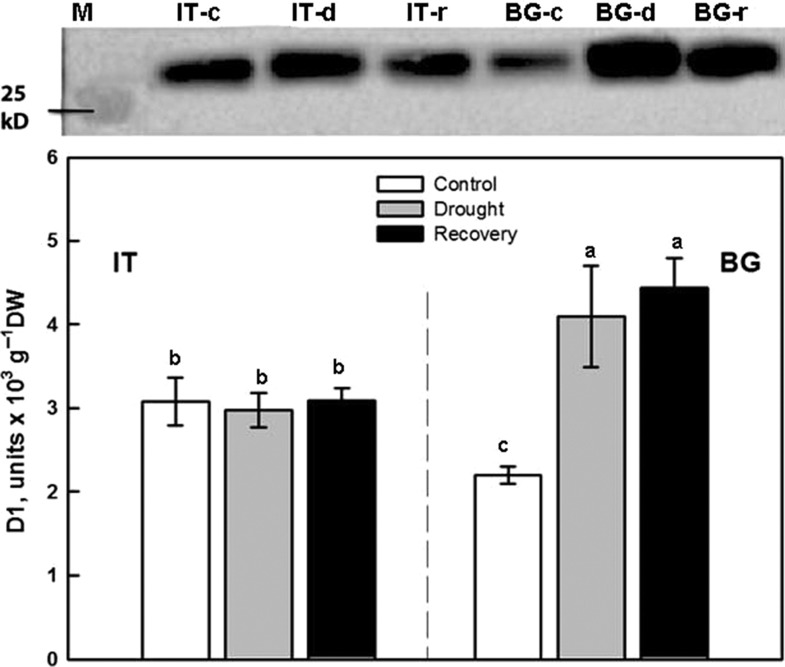
Western blot analysis and densitometric analysis of D1 protein in Italian (IT) and Bulgarian (BG) populations of *P. orientalis* plants under control conditions (95% FTSW) (white bars), during drought (28% FTSW) (gray bars) and after re-watering (90%-R FTSW) (black bars). The bar diagrams represent pixel volumes of D1 proteins in samples. The bands were normalized to the appropriate Actin band. Each value represents the mean ± SE (*n* = 3). Data were subjected to one-way ANOVA followed by Tukey’s test and bars accompanied by different letters are statistically different (*P* < 0.05).

The level of D1 protein was significantly higher in IT than in BG under control conditions (Fig. 6). Drought stress and recovery from drought did not affect D1 abundance in the IT ecotype. In the BG ecotype, D1 level increased significantly during drought (+186%), and after re-watering (+201%) with respect to control.

**Figure 7:**
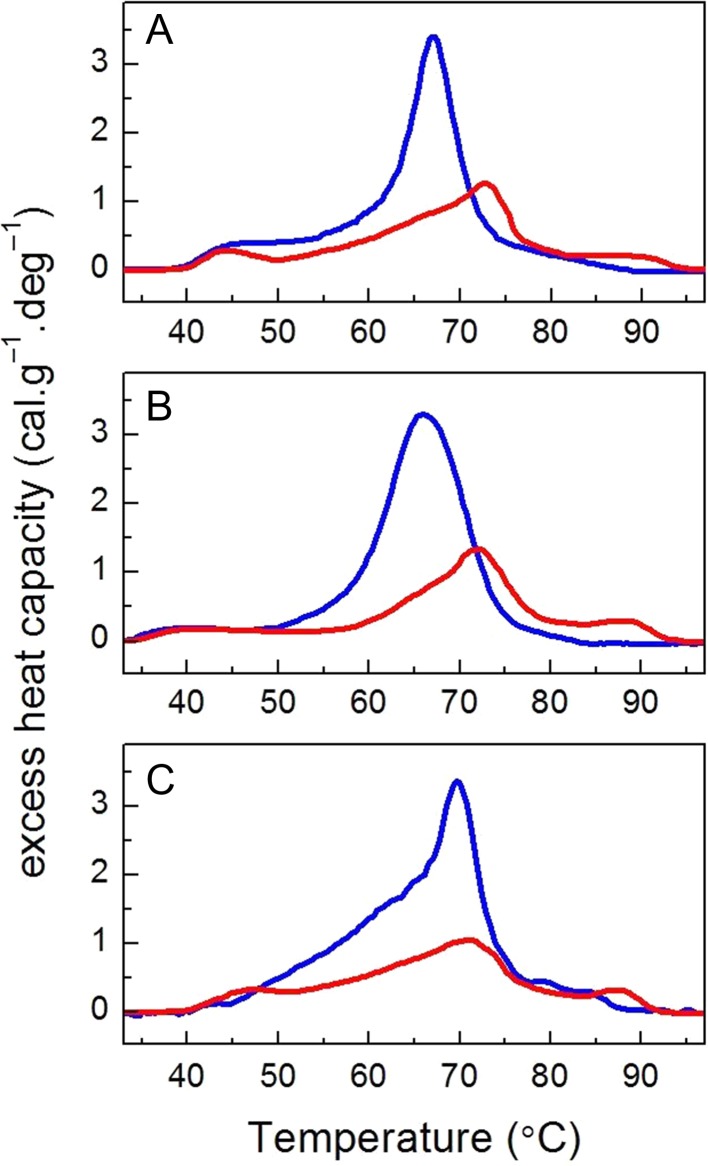
Representative DSC profiles recorded for thylakoid membranes isolated from IT (red lines) and BG (blue lines) populations of *P. orientalis* L. Control thermograms (**A**, 95% FTSW) are compared with those recorded for drought-stressed (**B**, 28% FTSW) and re-watered plants (**C**, 90-R% FTSW).

The calorimetric scans recorded for thylakoid membranes isolated from control BG and IT plants are presented in Fig. 7 and the derived calorimetric parameters are compared in Table 1. The excess heat capacity curves (thermograms) show that the photosynthetic complexes denatured in a narrow temperature interval and the midpoint temperatures of the major transitions were peaking at 73°C in the IT ecotype and at 67°C in the BG ecotype (Fig. 7, Table 1). Although the thermograms of the two ecotypes differed significantly both in the position of the main peak as well as in the enthalpy, the drought stress and re-watering did not change significantly those thermodynamic parameters for the two еcotypes (Table 1).

**Table 1:** Calorimetric parameters determined for thylakoid membranes isolated from the BG and IT ecotypes—transition temperatures (*T*_m_) of the sequential thermal transitions, total enthalpy (Δ*H*) and cooperativity (*T*_1/2_) of the thermogram. The plants were either grown in control conditions, subjected to drought stress and/or subsequently re-hydrated. Mean ± SD (*n* = 3). Data were subjected to one-way ANOVA followed by Tukey’s test. Means in the same row that are statistically different are shown by different letters (*P* < 0.05).

	IT			BG		
Treatment	Control	Drought	Re-watering	Control	Drought	Re-watering
Parameter						
*T*_m1_ (°C)	44.0 ± 2.0^**a**^	44.8 ± 2.8^**a**^	46.9 ± 1.8^**a**^	43.0 ± 2.8^**a**^	42.4 ± 2.8^**a**^	41.5 ± 2.1^**a**^
*T*_m2_ (°C)	72.7 ± 0.3^a^	70.9 ± 1.4^**a**^	70.9 ± 0.9^**a**^	67.2 ± 1.2^**b,c**^	65.5 ± 0.7^**c**^	70.0 ± 2.0^**a,b**^
*T*_m3_ (^o^C)	88.7 ± 0.8^**a**^	87.9 ± 0.1^**a**^	87.3 ± 1.1^**a**^			
Δ*H* (cal/g)	17.6 ± 7.5^**b**^	24.2 ± 2.5^**b**^	23.6 ± 2.3^**b**^	36.4 ± 1.6^**a**^	40.47 ± 0.8^**a**^	45.9 ± 2.8^**a**^

Since the thermal stability of membrane proteins strongly depends on their lipid environment we further explored the properties of the lipophilic marker MC540. The fluorescence bands peaking at 566 and 536 nm originate from MC540 molecules incorporated into loosely packed (fluid) and more tightly packed membrane domains, respectively. Thus the ratio *E*_566_/*E*_536_ reflects how fluid the thylakoid membranes are (Krumova et al., 2008). The two *Platanus* ecotypes showed similar *E*_566_/*E*_536_ ratio in control conditions (Table 2). Drought stress led to an increase of *E*_566_/*E*_536_ ratio in both ecotypes, while re-watering restored the *E*_566_/*E*_536_ ratio to the values of corresponding controls, thus indicating that the lipid packing in the thylakoid membranes of the two ecotypes was very similar.

**Table 2.** Fluorescence intensity ratio *E*_566_/*E*_536_ determined for MC_540_ incorporated in control, drought-stressed and re-watered thylakoid membranes of leaves of BG and IT ecotypes. Mean ± SD (*n* = 3). Data were subjected to one-way ANOVA followed by Tukey’s test and values accompanied by different letters are statistically different (*P* < 0.05).

	IT			BG		
Treatment	Control	Drought	Re-watering	Control	Drought	Re-watering
Parameter						
*E*_566_/*E*_536_ ratio	1.28 ± 0.07^**b**^	1.61 ± 0.08^**a**^	1.39 ± 0.10^**a,b**^	1.24 ± 0.09^**b**^	1.48 ± 0.07^**a,b**^	1.28 ± 0.11^**b**^

### Thermoluminescence

**Figure 8:**
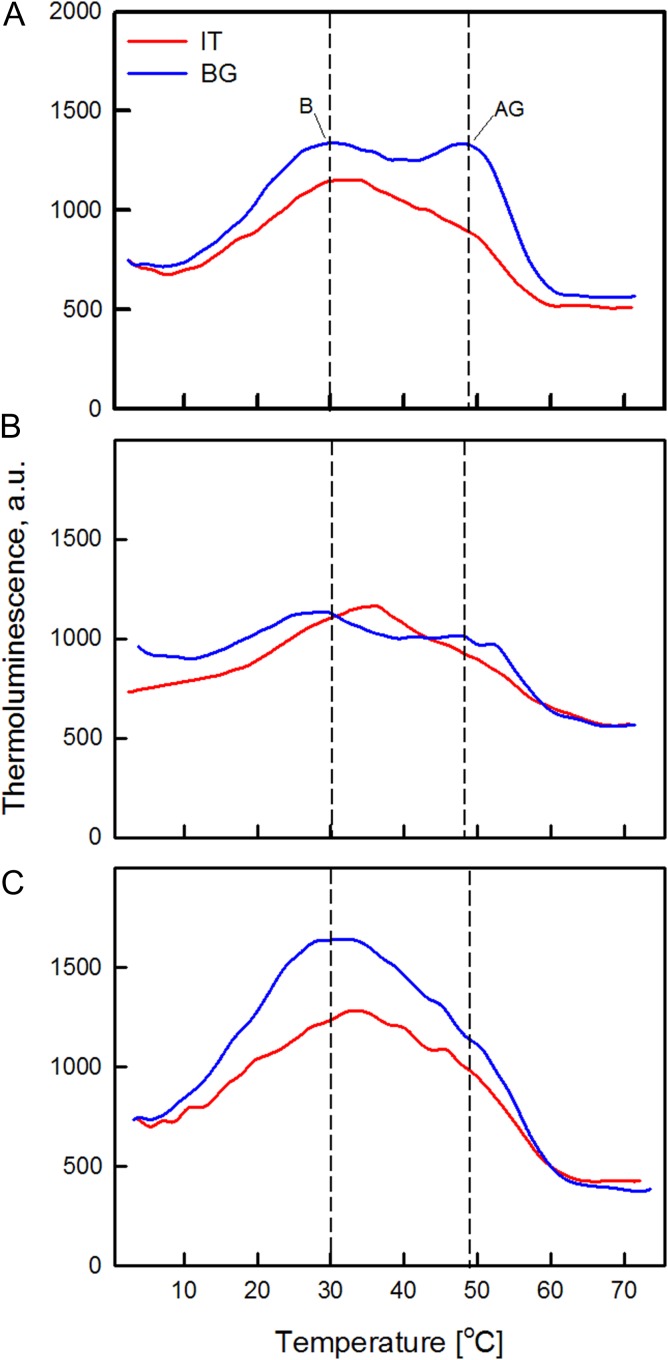
Representative thermoluminescence emission curves of control (**A**, 95% FTSW), drought-stressed (**B**, 28% FTSW) and re-watered (**C**, 90%-R FTSW) freshly excised leaf discs after excitation by two (2F) saturating xenon flashes. Prior to the measurements *Platanus orientalis* leaves of IT (red line) and BG (blue line) populations were dark-adapted for 4 hours. TL was recorded during heating of the samples up to 70°C at a rate of 0.5°C s^−^^1^.

In order to assess alterations of PSII primary photochemistry, TL emissions after flash(es) excitation were recorded (Fig. 8). In our case, we especially inspected the B band generated by *S*_2/3_ and *Q*_B_^−^ charge recombination (*S*_2/3_*Q*_B_^−^), and the so called “afterglow” (AG) band formed when an electron is back-transferred from stroma reductants to reduce the *Q*_B_ (*S*_2/3_*Q*_B_+e^−^) (Ducruet, 2003). No significant differences were detected in the maximum temperature values of B band in control leaves of the two ecotypes. After excitation by two single turnover flashes (2F) which produce the maximal overall emission, the main B band was peaking at around 30°C in both BG and IT (Fig. 8A). The intensity of the AG band which appeared as a shoulder near 48°C, was higher in BG than in IT controls (Fig. 8A), suggesting a higher cyclic electron flow in BG. In drought-stressed plants, TL emission decreased compared to controls, especially in the BG ecotype (Fig. 8B). This was accompanied by a statistically significant shift of the B band of the BG ecotype from 30.3°C ± 1.2°C (control) to 26.7°C ± 1.0°C (drought-stressed). No relevant changes in the B band position of the IT ecotype were observed, but the band was slightly upshifted (from 32.1°C ± 1.4°C in control to 34.3°C ± 1.5°C in drought-stressed leaves). Drought stress reduced the AG intensity in the BG ecotype, and again no effect in IT plants was detected. After re-watering the temperature of B band returned similar to controls (30.6°C ± 1.0°C and 33.4°C ± 1.0°C in BG and IT leaves, respectively) (Fig. 8C). The AG band almost disappeared in re-watered BG and IT leaves.

## Discussion

### *Platanus* *orientalis* ecotypes display specific physiological, structural and functional differences under control conditions

Physiological, structural and functional traits of *P. orentalis* plants originating from climatically different habitats were compared in order to assess the effect of local microclimate on plant adaptation.

No important differences in photosynthesis were found in control plants of the two ecotypes (Fig. 2). However, the ecotypes were different in their stomatal behavior (Fig. 3). Effective stomatal control is a fundamental eco-physiological trait for plant survival and adaptation to unfavorable environmental conditions allowing for optimal CO_2_-uptake and water-loss balance over a range of favorable and sub-optimal growth conditions (Raschke, 1975a; Cowan, 1978; Schulze and Hall, 1982; Hetherington and Woodward, 2003), helping plants to respond to and resist adverse environmental conditions (Haworth et al., 2011). It is interesting to note that only IT plants exhibited clear active stomatal behavior following the increases in [CO_2_] under control conditions (Fig. 3A). It is already demonstrated that stomata can be sensitized to CO_2_ by raising the level of ABA in the leaf, or by supplying ABA through the transpiration stream (Raschke, 1975b). We speculate that the active stomatal control in IT plants could be related to the more active plastidial methyl erythrol phosphate (MEP) pathway via which volatile and non-volatile isoprenoids, (including ABA, the hormone controlling active stomata opening) are synthesized (Lichtenthaler, 1999). Indeed, the IT ecotype is characterized by significantly higher isoprene emission than the BG ecotype. We recently demonstrated that in the isoprene-emitting *Arundo donax* the leaf level of ABA was much higher than in the non-emitting *Hakonechloa macra* under well-watered conditions (Velikova *et al.*, 2016), confirming that isoprene may proxy foliar ABA biosynthesis (Barta and Loreto, 2006). However, we cannot rule out any epigenetic modifications in IT ecotype due to the harsher environment in the place of origin, which could determine the active stomatal behavior of this ecotype.

No statistically significant difference in *F*_v_/*F*_m_ was found in control plants, suggesting similar efficiency of PSII, when all reaction centers were opened (Fig. 1). However, BG leaves that have lower isoprene emission exhibited significantly lower *Φ*_PSII_ compared with IT, indicating that a smaller fraction of the absorbed light energy was used for photochemistry. Similar to our observation, *Φ*_PSII_ was lower in non-isoprene emitting than in isoprene-emitting poplars (Velikova et al., 2015) and these results were correlated with down-regulation of the proteins involved in the photochemistry of photosynthesis (Velikova et al., 2014). Lower isoprene and *Φ*_PSII_ of BG plants were mirrored by a higher NPQ compared to IT leaves. The onset of this protective mechanism for the dissipation of excess excitation energy (Demmig-Adams and Adams, 2006) suggests that BG plants need to dissipate more absorbed energy as heat to protect their thylakoid membranes from photodamage (Murata et al., 2012). NPQ increase was already reported in plants with genetically down-regulated isoprene emission (Behnke et al., 2009; Velikova et al., 2015), and in non-emitters of the Arundinoideae subfamily when compared to natural isoprene emitters (Velikova *et al.*, 2016). Why high isoprene emitters do not need to dissipate non-radiatively as much energy as low or non-emitters? Many experiments indicate that isoprene stabilizes cellular membranes (Singsaas et al., 1997; Velikova et al., 2011, 2012), and smoothness linear electron flow between photosystems in the thylakoids (Pollastri et al., 2014). More recently, the significance of isoprene in structural organization of plastids, the chloroplast proteome profiling as well as the lipid matrix of thylakoid membranes has been demonstrated in genetically modified poplars with suppressed isoprene synthase (Velikova et al., 2014, 2015). In our experiment, the inner membrane system of IT mesophyll chloroplasts was better developed, comprising higher grana stacks than in BG chloroplasts (Fig. 5). A direct effect on the thylakoid membrane organization could be exerted by protein stoichiometry (Pribil et al., 2014), in particular, by PSII and LHCII assembly into supercomplexes and megacomplexes (Kouřil et al., 2012). Indeed, suppression of isoprene biosynthesis was accompanied by a reduction of the concentrations of the photosystem I and II reaction centers (Velikova et al., 2015), PsbP and PsbQ subunits of PSII, and the major light harvesting complexes of the two photosystems (Velikova et al., 2014). In plant mutants lacking PsbO and/or PsbP, a strong decrease in D1 and D2 protein content was demonstrated (Bricker and Frankel, 2011). We speculate that significantly lower level of D1 protein in BG ecotype could be related to altered protein profile and different structural organization of the thylakoid membrane system, compared to the IT ecotype. Indeed, TEM analyses illustrate that in BG chloroplasts the thylakoid membrane system consists of a relatively smaller number of grana thylakoids uniformly occupying the stroma space (compare Fig. 5A and 5D). Differences in the D1 protein among the two *Platanus* ecotypes (Fig. 6) might affect both the functionality and the structural organization of the photosynthetic apparatus.

To probe the conformational stability of the photosynthetic complexes (crucial for their functionality) and also to check how their thermal stability is affected by the different grana ultrastructure of the IT and BG ecotypes, a DSC analysis was carried out. Importantly, DSC allows measurements to be performed on native membranes and therefore takes into account the specific lipid–protein and protein–protein interactions. Previous studies on thylakoid membranes of higher plants (barley, spinach, pea) as well as on isolated sub-membrane fractions revealed multiple thermal transitions that can be ascribed to the denaturation of specific components of the photosynthetic apparatus and to the heat-induced disassembly of their lateral arrangement of the photosynthetic complexes (reviewed in Krumova et al., 2010). In contrast to those measurements, the thermograms recorded for IT and BG thylakoids in control conditions showed only one well defined transition along with 1 or 2 less defined transitions/shoulders (Fig. 7, Table 1). Therefore, we cannot discriminate the contribution of the multitude of photosynthetic proteins in *Platanus* thylakoid membranes to the denaturation transitions, and the dominant thermal transition can only be interpreted as representative of the general thermal stability of the thylakoid proteins. The shoulder at 44°C in the IT and BG thermograms can be attributed to heat-induced disorganization of the membrane affecting its lateral (macroorganization) and vertical (stacking) order, as already demonstrated for barley thylakoids (Dobrikova et al., 2003). It should be also noted that the IT ecotype exhibited significantly higher thermal transition temperature of the dominant peak (*T*_m2_) and nearly twice higher enthalpy (Table 1) than the BG ecotype. The higher stability of IT thylakoids might be due to the larger grana observed for this ecotype (Fig. 5A) or to altered physical properties of the lipid matrix that exert stabilizing effect on the photosynthetic proteins. Arguably, this may be associated to a larger presence of isoprene in IT thylakoids, and to the beneficial effect this compound exerts on the assembly and function of membranes (Pollastri et al., 2014; Velikova et al., 2015). The origin of the “smeared” high-temperature transition above 75°C is unclear and will not be discussed further in this work.

To explore the differences in the physical properties of the lipid matrix between the two populations we utilized the lipophilic fluorescent probe Merocyanine 540 (MC540). The incorporation of MC540 in thylakoid membranes was shown to be sensitive to the lipid packing (Krumova et al., 2008) and the presence of different lipid environments (Garab et al., 2017). The data presented on Table 2 strongly suggest that there is no change in the lipid phase behavior (as estimated by the *E*_566_/*E*_536_ ratio) between the two *Platanus* ecotypes under control conditions.

TL flash sequence experiments performed on dark-adapted controls exhibited typical oscillation pattern with a maximal intensity reached after two flashes, as normally observed for fully functional PSII (Fig. S1), and the emission decreased after three flashes. TL is associated mainly with the operation of PSII, the system that oxidizes water to oxygen and reduces plastoquinone (Demeter and Govindjee, 1989). Charge recombination in the oxygen evolving complex and reduced primary (*Q*_A_) or secondary (*Q*_B_) quinone electron acceptors of PSII contribute to the generation of TL bands (*Q* and *B*), and small changes in the redox properties of the radical pairs could affect the TL characteristics (Demeter and Govindjee, 1989). Thus, it could be expected that the observed significant differences in D1 protein amount in IT and BG chloroplasts will also influence TL emissions. However, no important alterations in the main B band were found. It was suggested that *S*_2_*Q*_A_^−^ stabilization by depletion of the 33-kDa protein could be due to modifications in the redox potentials of both *S*_2_ and *Q*_B_^−^, which compensate each other and result in an almost unchanged redox span of *S*_2_*Q*_B_^−^ (Demeter and Govindjee, 1989). Interestingly, the intensity of the AG band which appeared as a shoulder near 46°C, was higher in BG than in IT controls (Fig. 8A), while its position was almost unchanged, indicating the enhanced capacity of cyclic electron flow in BG samples (Ducruet, 2003). Cyclic electron flow is driven by photosystem I (PSI) in the light, and its activation may correspond to an increased demand in ATP, for protein synthesis or for other tolerance mechanisms which is fulfilled by cyclic electron flow (Bukhov and Carpentier, 2004). It is reported that cyclic flow contributes to pumping protons into the lumen, thus producing a stronger NPQ to dissipate the excess light energy (Cardol et al., 2003). Indeed, in our study NPQ was considerably higher in BG leaves compare to IT, even at control conditions.

### *Platanus* *orientalis* ecotypes respond differently to drought and to drought recovery

Drought caused significant reduction of photosynthesis in both BG and IT populations, accompanied by stomatal closure. Generally, diffusive resistances limit CO_2_ entry and photosynthesis under stress (Medrano et al., 2002; Flexas et al., 2008, 2016). However, in BG plants the intercellular [CO_2_] did not change, indicating availability of substrate for photosynthesis (Fig. 2C). Thus, biochemical and/or photochemical constraints are also responsible for photosynthesis inhibition in this ecotype. iWUE appears to be a plastic phenotype trait (Ackerly et al., 2000). Plants from xeric areas have higher iWUE than those from mesic areas only under drought conditions, and improved iWUE is the product of a gene x environment interaction contingent on the presence of a water deficit (Kooyers, 2015). Indeed, in our experiment significant difference in iWUE between ecotypes was observed only under drought stress. Interestingly, drought-induced significant increase of iWUE only in IT population, suggesting that IT leaves possess traits allowing better adaptation to drought than BG leaves. For example, the stomatal responses to [CO_2_] revealed active stomatal behavior only in IT plants exposed to drought. Interestingly, BG leaves acquired active stomatal response to CO_2_ after re-watering. Similar observations were reported in other species. In particular, it was shown that water deficit (Raschke, 1975b) and pre-chilling (Drake and Raschke, 1974) can sensitize stomata to CO_2_. The importance of effective stomatal control under both optimal growing conditions and photosynthetic constraints is amply reviewed (Cowan, 1978; Farquhar and Sharkey, 1982; Cornic, 2000; Chaves et al., 2003; Hetherington and Woodward, 2003), and suggests that plants with more active stomatal behavior will be more successful in unfavorable environments than species with less responsive stomatal control (Haworth et al., 2011). Acquisition of active stomatal control after recovering from the drought stress seems to be independent on foliar ABA, as isoprene level remained low in re-watered BG leaves. Perhaps ABA imported from the root via the classic xylematic transport of this hormone (Zhang and Davies, 1990) is responsible for sensitizing stomata of BG leaves to CO_2_ after recovering from drought.

The photochemistry of photosynthesis was also less affected by drought in IT than in BG leaves as indicated by chlorophyll fluorescence parameters. The higher *Φ*_PSII_ in drought-stressed IT compared to BG ecotype was not accompanied by higher net photosynthetic rate, suggesting increasing use of PSII electron flow for oxygenation of RuBP, i.e. to photorespiration. Indeed, *C*_i_ was significantly lower in IT than in BG leaves, indicating a different ratio between Rubisco substrates (CO_2_ and O_2_) in the mesophyll, and an increased partitioning of electron transport toward the photosynthetic oxidation cycle driving photorespiration (Figs 1C and 2C).

Photosynthesis did not recover completely in either of the investigated *P. orientalis* ecotypes. This observation confirms sensitivity of this mesophytic plant to drought, and shows that more intense and more frequently occurring future drought episodes (IPCC 2014) may severely endanger *P. orientalis*, impairing physiology and growth. Incomplete recovery of *A*_n_ could be mainly due to significantly lower *g*_s_ in IT ecotype. However, in BG plants *g*_s_ was restored to control value, while *A*_n_ did not recover, suggesting involvement of biochemical limitations, as also indicated by stability of intercellular [CO_2_] (Fig. 2C). Photochemical limitations affecting the incomplete recovery of net photosynthesis are also possible what is obvious from the fact that *F*_v_/*F*_m_ and *Φ*_PSII_ are lower in the BG ecotype after re-watering, compared to the corresponding controls (Fig. 1B and C). Moreover, as indicated by the data on Fig. 6 the BG ecotype responds by increased production of D1 protein which is attributed to active repair and *de novo* synthesis, which also supports the photochemical limitation due to stress-induced damages of the light harvesting mechanisms and reaction centers.

Isoprene emission of drought-stressed leaves decreased significantly only in the BG ecotype, while it remained almost unchanged in the IT ecotype. Under stress, the reduction in isoprene emission is predominantly due to limitation in carbon and energy supply through photosynthesis (Brilli et al., 2007; Tattini et al., 2014). In the present study, the inhibition of isoprene emission of drought-stressed leaves was associated with more pronounced alteration in the chloroplast ultrastructure in BG than in IT plastids. Our observations are consistent with previous studies with transgenic poplar (Velikova et al., 2015). Similar correlation between isoprene internal concentration and structural organization of thylakoid membranes was also found in two Arundinoideae species as stronger destructive alterations of the plastid membrane system were found in the species (*Hakonechloa macra*) which does not produce isoprene than the isoprene-emitting *Arundo donax* (Velikova *et al.*, 2016).

The D1 protein expression of the IT ecotype under drought remained similar to control conditions, confirming the stability of thylakoid membranes and PSII complexes. Previous studies have indicated that drought stress adversely affects the levels of thylakoid membrane proteins (Yuan et al., 2005; Liu et al., 2009; Chen *et al.*, 2016). However, drought stress promoted significant increase in the level of D1 protein in BG ecotype, which we interpret as a compensation mechanism for the reduced photochemistry observed in these drought-stressed plants. It was suggested that the increased accumulation of low molecular weigh antioxidants (Havaux et al., 2005; Ramel et al., 2012; Demmig-Adams et al., 2013) and increased activity of ROS-scavenging enzymes (Al-Taweel et al., 2007; Kornyeyev et al., 2003) might reduce the levels of intracellular ROS, thereby allowing the synthesis of D1 protein (Chen *et al.*, 2016). The functional and structural integrity of PSII is maintained by multi-step processes (for review see Theis and Schroda, 2016). Giardi *et al.* (1996) provided strong evidences that drought increases the extent of phosphorylation of the PSII core proteins and D1 protein synthesis in pea plants. It is reasonable to suppose that drought stress induces structural and functional reorganization of PSII in the BG ecotype. In particular, the significantly higher level of D1 in BG samples during drought and after re-watering might be due to enhanced PSII protein phosphorylation, and to faster turnover of D1 protein.

In our study, the drought-stressed plants of both ecotypes where characterized by higher *E*_566_/*E*_536_ ratio (Table 2). This indicates a more fluid lipid phase in the membranes (Krumova et al., 2008) that is probably related to the formation of lipid peroxidation products as evidenced by hexenal production (Fig. 4B). These changes, however, did not affect significantly the thermal stability of the thylakoid membranes as they were observed in both ecotypes. Upon re-watering the *E*_566_/*E*_536_ ratio decreased to values close to the control ones, again corresponding to a reduced hexenal level in re-watered plants, compared to drought-stressed plants (Fig. 4B and Table 2). This indicates that the two ecotypes are able to restore the physical properties of the lipid matrix upon re-watering.

TL revealed significant differences between ecotypes upon drought-stress occurrence. Drought stress induced a significant shift of B band to lower temperature only in the BG ecotype, indicating that the part of the PSII centers were destabilized by drought in these plants (Peeva and Maslenkova, 2004; Bürling et al., 2014). The higher stability of IT thylakoid membranes positively correlated with increased isoprene emission, which increased only in IT drought-stressed samples. This supports the idea that isoprene helps stabilize membrane properties (Velikova et al., 2011; Ahrar et al., 2015). However, the temperature maximum of B band returned to the value similar to control after re-watering, indicating no permanent impairment of PSII in recovering BG plants. The intensity of the AG band is also often found to increase in dehydrated leaves (Peeva et al., 2012; Bürling et al., 2014). However, AG intensity decreased in drought-stressed BG ecotype, and almost disappeared after re-watering, suggesting low capacity of the biochemical machinery to support the cyclic and chlororespiratory pathways (Bürling et al., 2014). On the contrary, no changes in AG band intensity and position were observed in IT leaves.

In summary, the results of this study confirmed that *P. orientalis* plants are inherently sensitive to drought, but also revealed that ecotypes originating from harsher (drier) environments possess physiological and structural traits that could help them better overcome short-term drought-stress events. Our study suggests that isoprene plays a role in plant adaptation to drought. Aside its direct antioxidant action (Vickers et al., 2009) we surmise that the higher isoprene emission (hence production) in drier environments at the edge of *P. orientalis’*s habitat (e.g. Sicily, Italy), may affect in chloroplast fine structure, the physical properties of thylakoid membranes, and overall functionality of the photosynthetic apparatus, conductive to a more efficient utilization of absorbed light energy for photochemistry, and less damage to photosynthesis. Although a short-term drought-stress episode did not cause any irreversible changes in the photosynthetic machinery of either ecotype, the functional and structural traits observed in the IT ecotype may be key for supporting resistance, resilience, and adaptation to drought stress, conditions expected to be more widespread and more frequently recurring in the *P. orientalis* habitat in the future.

Ultimately, in the context of climate change, it is essential to understand how fast variations of the environment will modify the adaptive capacity of relict species, especially trees. While ecotypes adapted to dry and warm environments will better resist/be resilient better to climate extremization (as shown here), more studies on the relationships between adaptation strategies, genetic and epigenetic diversity, and population structure are needed for developing *in situ* and *ex situ* conservation strategies (Frankel et al., 1995).

## Supplementary Material

Supplementary Data

## References

[coy073C1] Ackerly DD, Dudley SA, Sultan SE, Schmitt J, Coleman JS, Linder CR, Sandquist DR, Geber MA, Evans AS, Dawson TE, et al (2000) The evolution of plant ecophysiological traits: recent advances and future directions. BioScience 50: 979–995.

[coy073C2] Ahrar M, Doneva D, Koleva D, Romano A, Rodeghiero M, Tsonev T, Biasioli F, Stefanova M, Peeva V, Wohlfahrt G, et al (2015) Isoprene emission in the monocot *Arundineae* tribe in relation to functional and structural organization of the photosynthetic apparatus. Environ Exp Bot 119: 87–95.

[coy073C3] Ahrar M, Doneva D, Tattini M, Brunetti C, Gori A, Rodeghiero M, Wohlfahrt G, Biasioli F, Varotto C, Loreto F, et al (2017) Phenotypic differences determine drought stress responses in ecotypes of *Arundo donax* adapted to different environments. J Exp Bot 168: 2439–2451. 10.1093/jxb/erx125 28449129

[coy073C4] Aitken SN, Yeaman S, Holliday JA, Wang T, Curtis-McLane S (2008) Adaptation, migration or extirpation: climate change outcomes for trees populations. Evol Appl 1: 95–111. 25567494 10.1111/j.1752-4571.2007.00013.x PMC3352395

[coy073C5] Al-Taweel K, Iwaki T, Yabuta Y, Shigeoka S, Murata N, Wadano A (2007) A bacterial transgene for catalase protects translation of d1 protein during exposure of salt-stressed tobacco leaves to strong light. Plant Physiol 145: 258–265. 17660354 10.1104/pp.107.101733 PMC1976566

[coy073C6] Arena C, Figlioli F, Sorrentino MC, Izzo LG, Capozzi F, Giordano S, Spagnuolo V (2017) Ultrastructural, protein and photosynthetic alteration induced by Pb and Cd in *Cynara cardunculus* L. and its potential for phytoremediation. Ecotoxicol Environ Saf 145: 83–89. 28708985 10.1016/j.ecoenv.2017.07.015

[coy073C7] Arnon DI (1949) Copper enzymes in isolated chloroplasts. Polyphenol oxidase in *Beta vulgaris*. Plant Physiol 24: 1–15. 16654194 10.1104/pp.24.1.1 PMC437905

[coy073C8] Barstow M, Rivers MC (2017) *Platanus orientalis*. The IUCN Red List of Threatened Species.

[coy073C9] Barta C, Loreto F (2006) The relationship between themethyl-erythritol phosphate pathway leading to emission of volatile isoprenoids and abscisic acid content in leaves. Plant Physiol 141: 1676–1683. 16766667 10.1104/pp.106.083063 PMC1533953

[coy073C10] Beckett M, Loreto F, Velikova V, Brunetti C, Di Ferdinando M, Tattini M, Calfapietra C, Farrant JM (2012) Photosynthetic limitations and volatile and non-volatile isoprenoids in the poikilochlorophyllous resurrection plant *Xerophyta humilis* during dehydration and rehydration. Plant Cell Environ 35: 2061–2074. 22582997 10.1111/j.1365-3040.2012.02536.x

[coy073C11] Becklin KM, Anderson JT, Gerhart LM, Wadgymar SM, Wessinger CA, Ward JK (2016) Examining plant physiological responses to climate change through an evolutionary lens. Plant Physiol 172: 635–649. 27591186 10.1104/pp.16.00793 PMC5047093

[coy073C12] Behnke K, Kleist E, Uerlings R, Wildt J, Rennenberg H, Schnitzler JP (2009) RNAi-mediated suppression of isoprene biosynthesis in hybrid poplar impacts ozone tolerance. Tree Physiol 29: 725–736. 19324699 10.1093/treephys/tpp009

[coy073C13] Bilger W, Björkman O (1991) Temperature dependence of violaxanthin de-epoxidation and non-photochemical fluorescence quenching in intact leaves of *Gossypium hirsutum* L. and *Malva parviflora* L. Planta 184: 226–234. 24194074 10.1007/BF00197951

[coy073C14] Blum A (1996) Crop responses of drought and the interpretation of adaptation. Plant Growth Regul 20: 135–148.

[coy073C15] Bricker TM, Frankel LK (2011) Auxiliary functions of the PsbO, PsbP and PsbQ proteins of higher plant photosystem II: a critical analysis. J Photochem Photobiol, B: Biology 104: 165–178. 21353792 10.1016/j.jphotobiol.2011.01.025

[coy073C16] Brilli F, Barta C, Fortunati A, Lerdau M, Loreto F, Centritto M (2007) Response of isoprene emission and carbon metabolism to drought in white poplar (*Populus alba*) saplings. New Phytol 175: 244–254. 17587373 10.1111/j.1469-8137.2007.02094.x

[coy073C17] Bukhov N, Carpentier R (2004) Alternative photosystem I-driven electron transport routes: mechanisms and functions. Photosynth Res 82: 17–33. 16228610 10.1023/B:PRES.0000040442.59311.72

[coy073C18] Bürling K, Ducruet J-M, Cornic G, Hunsche M, Cerovic ZG (2014) Assessment of photosystem II thermoluminescence as a tool to investigate the effects of dehydration and rehydration on the cyclic/chlororespiratory electron pathways in wheat and barley leaves. Plant Sci 223: 116–123. 24767121 10.1016/j.plantsci.2014.03.013

[coy073C19] Cardol P, Gloire G, Havaux M, Remacle C, Matagne R, Franck F (2003) Photosynthesis and state transitions in mitochondrial mutants of *Chlamydomonas reinhardtii* affected in respiration. Plant Physiol 133: 2010–2020. 14630958 10.1104/pp.103.028076 PMC300752

[coy073C20] Caruso G, Gangale C, Uzunov D, Pignotti L (2008) Chorology of *Platanus orientalis* (Platanaceae) in Calabria (S Italy). Phytol Balcan 14: 51–56.

[coy073C21] Chaves MM, Flexas J, Pinheiro C (2009) Photosynthesis under drought and salt stress: regulation mechanisms from whole plant to cell. Ann Bot 103: 551–560. 18662937 10.1093/aob/mcn125 PMC2707345

[coy073C22] Chaves MM, Maroco JP, Pereira JS (2003) Understanding plant responses to drought—from genes to the whole plant. Funct Plant Biol 30: 239–264. 10.1071/FP02076 32689007

[coy073C23] Chaves MM, Oliveira MM (2004) Mechanisms underlying plant resilience to water deficits: prospects for water-saving agriculture. J Exp Bot 55: 2365–2384. 15475377 10.1093/jxb/erh269

[coy073C24] Chen YE, Liu WJ, Su YQ, Cui JM, Yuan M, Zhang HY, Yuan S (2016) Different response of photosystem II to short and long-term drought stress in *Arabidopsis thaliana*. Physiol Plant 158: 225–235. 26918860 10.1111/ppl.12438

[coy073C25] Cornic G (2000) Drought stress inhibits photosynthesis by decreasing stomatal aperture—not by affecting ATP synthesis. Trends Plant Sci 5: 187–188.

[coy073C26] Cowan IR (1978) Stomatal behaviour and environment. Adv Behav Environ 4: 117–228.

[coy073C27] David TS, Henriques MO, Kurz-Besson C, Nunes J, Valente F, Vaz M, Pereira JS, Siegwolf R, Chaves MM, Gazarini LC, et al (2007) Water use strategies in two co-occurring Mediterranean evergreen oaks: surviving the summer drought. Tree Physiol 27: 793–803. 17331898 10.1093/treephys/27.6.793

[coy073C28] Demeter S, Govindjee (1989) Thermoluminescence in plants. Physiol Plant 75: 121–130.

[coy073C29] Demmig-Adams B, Adams WW III (2006) Photoprotection in an ecological context: the remarkable complexity of thermal energy dissipation. New Phytol 172: 11–21. 16945085 10.1111/j.1469-8137.2006.01835.x

[coy073C30] Demmig-Adams B, Cohu CM, Amiard V, Zadelhoff G, Veldink GA, Muller O, Adams WW (2013) Emerging trade-offs impact of photoprotectants (PsbS, xanthophylls, and vitamin E) on oxylipins as regulators of development and defense. New Phytol 197: 720–729. 23418633 10.1111/nph.12100

[coy073C31] Dobrikova AG, Várkonyi Z, Krumova SB, Kovács L, Kostov GK, Todinova SJ, Busheva MC, Taneva SG, Garab G (2003) Structural rearrangements in chloroplast thylakoid membranes revealed by differential scanning calorimetry and circular dichroism spectroscopy. Thermo-optic effect. Biochemistry 42: 11272–11280. 14503877 10.1021/bi034899j

[coy073C32] Drake B, Raschke K (1974) Prechilling of *Xanthium strumarium* L. reduces net photosynthesis and, independently, stomatal conductance, while sensitizing the stomata to CO_2_. Plant Physiol 53: 808–812. 16658795 10.1104/pp.53.6.808 PMC541453

[coy073C33] Ducruet JM (2003) Chlorophyll thermoluminescence of leaf discs: simple instruments and progress in signal interpretation open the way to new ecophysiological indicators. J Exp Bot 54: 2419–2430. 14565948 10.1093/jxb/erg268

[coy073C34] Farquhar GD, Sharkey TD (1982) Stomatal conductance and photosynthesis. Ann Rev Plant Physiol 33: 317–345.

[coy073C35] Finni A, Tattini M, Esteban R (2017) Editorial: plant’s responses to novel environmental pressures. Front Plant Sci 8: 2000. 29218055 10.3389/fpls.2017.02000 PMC5704371

[coy073C36] Flexas J, Bota J, Loreto F, Cornic G, Sharkey TD (2004) Diffusive and metabolic limitations to photosynthesis under drought and salinity in C3 plants. Plant Biol 6: 269–279. 15143435 10.1055/s-2004-820867

[coy073C37] Flexas J, Díaz-Espejo A, Conesa MA, Coopman RE, Douthe C, Gago J, Gallé A, Galmés J, Medrano H, Ribas-Carbo M, et al (2016) Mesophyll conductance to CO_2_ and Rubisco as targets for improving intrinsic water use efficiency in C3 plants. Plant Cell Environ 39: 965–982. 26297108 10.1111/pce.12622

[coy073C38] Flexas J, Ribas-Carbу M, Díaz-Espejo A, Galmés J, Medrano H (2008) Mesophyll conductance to CO_2_: current knowledge and future prospects. Plant Cell Environ 31: 602–621. 17996013 10.1111/j.1365-3040.2007.01757.x

[coy073C39] Frankel OH, Brown AHD, Burdon JJ.(1995) The conservation of plant biodiversity. Cambridge University Press, Cambridge, New York, p 299.

[coy073C40] Garab G, Ughy B, de Waard P, Akhtar P, Javornik U, Kotakis C, Šket P, Karlický V, Materová Z, Špunda V, et al (2017) Lipid polymorphism in chloroplast thylakoid membranes—as revealed by 31P-NMR and time-resolved merocyanine fluorescence spectroscopy. Sci Rep 7: 13343 Art num. https://www.nature.com/articles/s41598-017-13574-y.pdf. 29042649 10.1038/s41598-017-13574-y PMC5645462

[coy073C41] Genty B, Briantais JM, Baker NR (1989) The relationship between the quantum yield of photosynthetic electron transport and quenching of chlorophyll fluorescence. Biochim Biophys Acta 990: 87–92.

[coy073C42] Giardi MT, Cona A, Geiken B, Kučera T, Masojídek J, Mattoo AK (1996) Long-term drought stress induces structural and functional reorganization of photosystem II. Planta 199: 118–125.

[coy073C43] Grueva M, Zhelev P (2011) Population genetic structure of *Platanus orientalis* L. in Bulgaria. iForest 4: 186–189.

[coy073C44] Harrison MA, Melis A (1992) Organization and stability of polypeptides associated with chlorophyll a–b light-harvesting complex of photosystem II. Plant Cell Physiol 33: 627–637.

[coy073C45] Havaux M, Eymery F, Porfirova S, Rey P, Dormann P (2005) Vitamin E protects against photoinhibition and photooxidative stress in *Arabidopsis thaliana*. Plant Cell 17: 3451–3469. 16258032 10.1105/tpc.105.037036 PMC1315381

[coy073C46] Haworth M, Elliott-Kingston C, McElwain C (2011) Stamatal control as a driver of plant evolution. J Exp Bot 62: 2419–2423. 21576397 10.1093/jxb/err086

[coy073C47] Haworth M, Killi D, Materassi A, Raschi A (2015) Coordination of stomatal physiological behavior and morphology with carbon dioxide determines stomatal control. Am J Bot 102: 677–688. 26022482 10.3732/ajb.1400508

[coy073C48] Hetherington AM, Woodward FI (2003) The role of stomata in sensing and driving environmental change. Nature 424: 901–908. 12931178 10.1038/nature01843

[coy073C49] Hoffmann AA, Sgro CM (2011) Climate change and evolutionary adaptation. Nature 470: 479–485. 21350480 10.1038/nature09670

[coy073C50] IPCC (2014) Climate change 2014: mitigation of climate change In Edenhofer OR, Pichs-Madruga Y, Sokona E, Farahani S, Kadner K, Seyboth A, et al, eds Contribution of Working Group III to the Fifth Assessment Report of the Intergovernmental Panel on Climate Change. Cambridge University Press, Cambridge.

[coy073C51] Kesselmeier J, Staudt M (1999) Biogenic Volatile Organic Compounds (VOC): an overview on emission physiology and ecology. J Atmos Chem 33: 23–88.

[coy073C52] Kooyers NJ (2015) The evolution of drought escape and avoidance in natural herbaceous populations. Plant Sci 234: 155–162. 25804818 10.1016/j.plantsci.2015.02.012

[coy073C53] Kornyeyev D, Logan BA, Allen RD, Holaday AS (2003) Effect of chloroplastic overproduction of ascorbate peroxidase on photosynthesis and photoprotection in cotton leaves subjected to low temperature photoinhibition. Plant Sci 165: 1033–1041.

[coy073C54] Kouřil R, Dekker JP, Boekema EJ (2012) Supramolecular organization of photosystem II in green plants. Biochim Biophys Acta 1817: 2–12. 21723248 10.1016/j.bbabio.2011.05.024

[coy073C55] Krumova SB, Koehorst RB, Bóta A, Páli T, van Hoek A, Garab G, van Amerongen H (2008) Temperature dependence of the lipid packing in thylakoid membranes studied by time- and spectrally resolved fluorescence of Merocyanine 540. Biochim Biophys Acta 1778: 2823–2833. 18929531 10.1016/j.bbamem.2008.09.007

[coy073C56] Krumova SB, Laptenok SP, Kovács L, Tóth T, van Hoek A, Garab G, van Amerongen H (2010) Digalactosyl-diacylglycerol-deficiency lowers the thermal stability of thylakoid membranes. Photosynth Res 105: 229–242. 20645128 10.1007/s11120-010-9581-5 PMC2975056

[coy073C57] Lawlor DW, Tezara W (2009) Causes of decreased photosynthetic rate and metabolic capacity in water-deficient leaf cells: a critical evaluation of mechanisms and integration of processes. Ann Bot 103: 561–579. 19155221 10.1093/aob/mcn244 PMC2707350

[coy073C58] Leimu R, Vergeer P, Angeloni F, Ouborg N (2010) Habitat fragmentation, climate change, and inbreeding in plants. Ann NY Acad Sci 1195: 84–98. 20536818 10.1111/j.1749-6632.2010.05450.x

[coy073C59] Lichtenthaler HK (1999) The 1-deoxy-d-xylulose-5-phosphate pathway of isoprenoid biosynthesis in plants. Ann Rev Plant Physiol Plant Mol Biol 50: 47–65. 15012203 10.1146/annurev.arplant.50.1.47

[coy073C60] Liu WJ, Chen YE, Tian WJ, Du JB, Zhang ZW, Xu F, Zhang F, Yuan S, Lin HH (2009) Dephosphorylation of photosystem II proteins and phosphorylation of CP29 in barley photosynthetic membranes as a response to water stress. Biochim Biophys Acta 1787: 1238–1245. 19409367 10.1016/j.bbabio.2009.04.012

[coy073C61] Loreto F, Barta C, Brilli F, Nogues I (2006) On the induction of volatile organic compound emissions by plants as consequence of wounding or fluctuations of light and temperature. Plant Cell Environ 29: 1820–1828. 16913871 10.1111/j.1365-3040.2006.01561.x

[coy073C62] Loreto F, Dicke M, Schnitzler J-P, Turlings TCJ (2014) Plant volatiles and the environment. Plant Cell Environ 37: 1905–1908. 24811745 10.1111/pce.12369

[coy073C63] Loreto F, Fineschi S (2015) Reconciling functions and evolution of isoprene emission in higher plants. New Phytol 206: 578–582. 25557381 10.1111/nph.13242

[coy073C64] Maroco JP, Pereira JS, Chaves MM (1997) Stomatal responses to leaf-to-air vapour pressure deficit in Sahelian species. Aust J Plant Physiol 24: 381–387.

[coy073C65] Medrano H, Escalona JM, Bota J, Gulías J, Flexas J (2002) Regulation of photosynthesis of C3 plants in response to progressive drought: the interest of stomatal conductance as a reference parameter. Ann Bot 89: 895–905. 12102515 10.1093/aob/mcf079 PMC4233802

[coy073C66] Monson RK, Jones RT, Rosenstiel TN, Schnitzler JP (2013) Why only some plants emit isoprene. Plant Cell Environ 36: 503–516. 22998549 10.1111/pce.12015

[coy073C67] Murata N, Allakhverdiev SI, Nishiyama Y (2012) The mechanism of photoinhibition *in vivo*: re-evaluation of the roles of catalase, α-tocopherol, non-photochemical quenching, and electron transport. Biochim Biophys Acta 1817: 1127–1133. 22387427 10.1016/j.bbabio.2012.02.020

[coy073C68] Ngugi MR, Doley D, Hunt MA, Ryan P, Dart P (2004) Physiological responses to water stress in *Eucalyptus cloeziana* and *E. argophloia* seedlings. Trees 18: 381–389.

[coy073C69] Nicotra AB, Atkin OK, Bonser SP, Davidson AM, Finnegan EJ, Mathesius U, Poot P, Purugganan MD, Richards CL, Valladares F, et al (2010) Plant phenotypic plasticity in a changing climate. Trends Plant Sci 15: 684–692. 20970368 10.1016/j.tplants.2010.09.008

[coy073C70] Peeva V, Maslenkova L (2004) Thermoluminescence study of photosystem II activity in *Haberlea rhodopensis* and spinach leaves during desiccation. Plant Biol 6: 319–324. 15143440 10.1055/s-2004-820873

[coy073C104] Peeva VN, Tóth SZ, Cornic G, Ducruet JM (2012) Thermoluminescence and P700 redox kinetics as complementary tools to investigate the cyclic/chlororespiratory electron pathways in stress conditions in barley leaves. Physiol Plant 144: 83--97. 144: 83–97. 10.1111/j.1399-3054.2011.01519.x 21910736

[coy073C71] Pollastri S, Tsonev T, Loreto F (2014) Isoprene improves photochemical efficiency and enchances heat dissipation in plants at physiological temperatures. J Exp Bot 65: 1565–1570. 24676032 10.1093/jxb/eru033 PMC3967094

[coy073C72] Pribil M, Labs M, Leister D (2014) Structure and dynamics of thylakoids in land plants. J Exp Bot 65: 1955–1972. 24622954 10.1093/jxb/eru090

[coy073C73] Ramel F, Birtic S, Cuine S, Triantaphylides C, Ravanat JL, Havaux M (2012) Chemical quenching of singlet oxygen by carotenoids in plants. Plant Physiol 158: 1267–1278. 22234998 10.1104/pp.111.182394 PMC3291260

[coy073C74] Raschke K (1975a) Stomatal action. An Rev Plant Physiol 26: 309–340.

[coy073C75] Raschke K (1975b) Simultaneous requirement of carbon dioxide and abscisic acid for stomatal closing in *Xanthium strumaruim* L. Planta 125: 243–259. 24435438 10.1007/BF00385601

[coy073C76] Rosati L, Masi A, Giardini M, Marignani M (2015) Under the shadow of a big plane tree: why *Platanus orientalis* should be considered an archaeophyte in Italy. Plant Biosyst 149: 185–194.

[coy073C77] Schulze E-D, Hall AE (1982) Stomatal responses, water loss and CO_2_ assimilation rates of plants In Lange OL, Nobel PS, Osmond CB, Ziegler H, eds Physiological Plant Ecology II. Water Relations and Carbon Assimilation. Springer-Verlag, Berlin Heidelberg New York, pp 181–230.

[coy073C78] Sharkey TD, Gray DW, Pell HK, Breneman SR, Topper L (2013) Isoprene synthase genes form a monophyletic clade of acyclic terpene synthases in the Tps-b terpene synthase family. Evolution 67: 1026–1040. 23550753 10.1111/evo.12013

[coy073C79] Sharkey TD, Yeh S (2001) Isoprene emission from plants. Annu Rev Plant Physiol Plant Mol Biol 52: 407–436. 11337404 10.1146/annurev.arplant.52.1.407

[coy073C80] Silim S, Nash R, Reynard D, White B, Schroder W (2009) Leaf gas exchange and water potential respones to droght in nine poplar (*Populus* spp.) clones with contrasting drought tolerance. Trees 23: 959–969.

[coy073C81] Singsaas EL, Lerdau M, Winter K, Sharkey TD (1997) Isoprene increases thermotolerance of isoprene-emitting species. Plant Physiol 115: 1413–1420. 12223874 10.1104/pp.115.4.1413 PMC158606

[coy073C82] Stockwell C, Hendry A, Kinnison M (2003) Contemporary evolution meets conservation. Trends Ecol Evol 18: 94–101.

[coy073C83] Tattini M, Loreto F, Fini A, Guidi L, Brunetti C, Velikova V, Gori A, Ferrini F (2015) Isoprenoids and phenylpropanoids are part of the antioxidant defense orchestrated daily by drought stressed *Platanus* x *acerifolia* plants during Mediterranean summers. New Phytol 207: 613–626. 25784134 10.1111/nph.13380

[coy073C84] Tattini M, Velikova V, Vickers C, Brunetti C, Di Ferdinando M, Trivellini A, Fineschi S, Agati G, Ferrini F, Loreto F (2014) Isoprene production in transgenic tobacco alters isoprenoid, nonstructural carbohydrate and phenylpropanoid metabolism, and protects photosynthesis from drought stress. Plant Cell Environ 37: 1950–1964. 24738622 10.1111/pce.12350

[coy073C85] Theis J, Schroda M (2016) Revising the photosystem II repair cycle. Plant Signal Behav 11: e1218587. 27494214 10.1080/15592324.2016.1218587 PMC5058467

[coy073C86] Theurillat JP, Guisan A (2001) Potential impact of climate change on vegetation in the European alps: a review. Clim Change 50: 77–109.

[coy073C87] Valladares F, Gianoli E, Gómez JM (2007) Ecological limits to plant phenotypic plasticity. New Phytol 176: 749–763. 17997761 10.1111/j.1469-8137.2007.02275.x

[coy073C88] Velikova V, Brunetti C, Tattini M, Doneva D, Ahrar M, Tsonev T, Stefanova M, Ganeva T, Gori A, Ferrini F, et al (2016) Physiological significance of isoprenoids and phenylpropanoids in drought response of *Arundinoideae* species with contrasting habitats and metabolism. Plant Cell Environ 39: 2185–2197. 27351898 10.1111/pce.12785

[coy073C89] Velikova V, Ghirardo A, Vanzo E, Merl J, Hauck S, Schnitzler J-P (2014) The genetic manipulation of isoprene emissions in poplar plants remodels the chloroplast proteome. J Proteome Res 13: 2005–2018. 24650239 10.1021/pr401124z

[coy073C90] Velikova V, Loreto F, Tsonev T, Brilli F, Edreva A (2006) Isoprene prevents the negative consequences of high temperature stress in *Platanus orientalis* leaves. Funct Plant Biol 33: 931–940. 10.1071/FP06058 32689303

[coy073C91] Velikova V, Müller C, Ghirardo A, Rock TM, Aichler M, Walch A, Schmitt-Kopplin P, Schnitzler J-P (2015) Knocking down isoprene emission modifies the lipid matrix of thylakoid membranes and influences the chloroplast ultrastructure in poplar. Plant Physiol 168: 859–870. 25975835 10.1104/pp.15.00612 PMC4741320

[coy073C92] Velikova V, Sharkey TD, Loreto F (2012) Stabilization of thylakoid membranes in isoprene-emitting plants reduces formation of reactive oxygen species. Plant Signal Behav 7: 139–141. 22301981 10.4161/psb.7.1.18521 PMC3357355

[coy073C93] Velikova V, Várkonyi Z, Szabó M, Maslenkova L, Nogues I, Kovács L, Peeva V, Busheva M, Garab G, Sharkey TD, et al (2011) Increased thermostability of thylakoid membranes in isoprene-emitting leaves probed with three biophysical techniques. Plant Physiol 157: 905–916. 21807886 10.1104/pp.111.182519 PMC3192565

[coy073C94] Vickers CE, Gershenzon J, Lerdau MT, Loreto F (2009) A unified mechanism of action for volatile isoprenoids in plant abiotic stress. Nat Chem Biol 5: 283–291. 19377454 10.1038/nchembio.158

[coy073C95] von Caemmerer S, Farquhar GD (1981) Some relationships between the biochemistry of photosynthesis and the gas exchange of leaves. Planta 153: 376–387. 24276943 10.1007/BF00384257

[coy073C96] Wang W, Vignani R, Scali M, Cresti M (2006) A universal and rapid protocol for protein extraction from recalcitrant plant tissues for proteomic analysis. Electrophoresis 27: 2782–2786. 16732618 10.1002/elps.200500722

[coy073C97] Yuan S, Liu W-J, Zhang N-H, Wang M-B, Liang H-G., Lin H-H (2005) Effects of water stress on major photosystem II gene expression and protein metabolism in barley leaves. Physiol Plant 125: 464–473.

[coy073C98] Zeinalov Y, Maslenkova L (1996) A computerized equipment for thermoluminescence investigations. Bulg J Plant Physiol 22: 88–94.

[coy073C99] Zhang J, Davies WJ (1990) Changes in the concentration of ABA in xylem sap as a function of changing soil water status can account for changes in leaf conductance and growth. Plant Cell Environ 13: 277–285, 1990.

