# Supplementary material for: Physiological and structural adjustments of two ecotypes of Platanus orientalis L. from different habitats in response to drought and re-watering
Source: Conserv Physiol. 2018 Dec 20;6(1):coy073. doi: 10.1093/conphys/coy073 (PMC6301291; doi:10.1093/conphys/coy073)
Supplement: Supplementary Data [file coy073_figure_s1.docx]

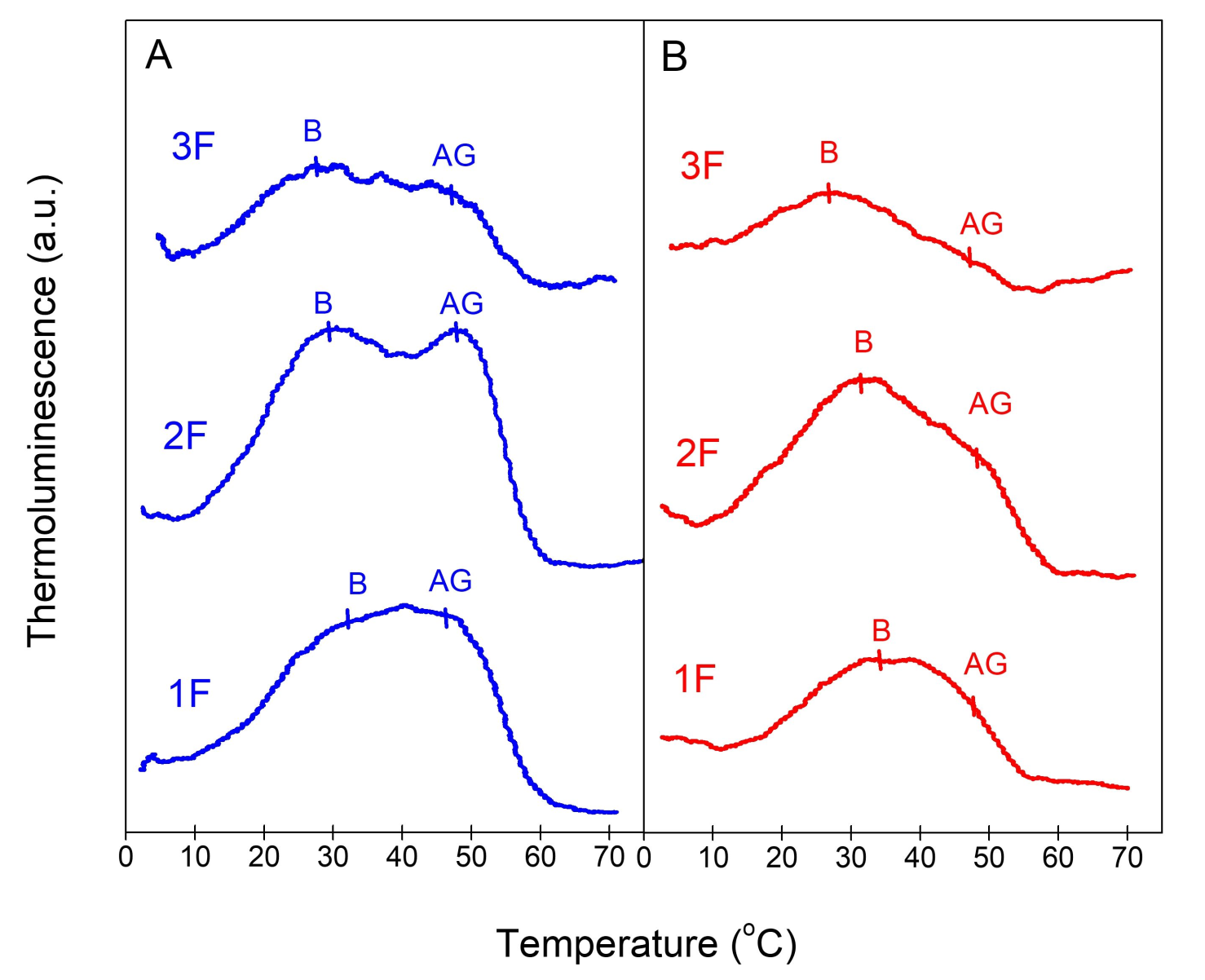


**Figure S1:** Thermoluminescence emissions from freshly excised leaf discs of 4 hours dark-adapted unstressed *Platanus* *orientalis* leaves (A – BG; B - IT ecotypes) after excitation by one (1F), two (2F) or three (3F) saturating xenon flashes
